# Autophagy and Tumor Database: ATdb, a novel database connecting autophagy and tumor

**DOI:** 10.1093/database/baaa052

**Published:** 2020-07-07

**Authors:** Kelie Chen, Dexin Yang, Fan Zhao, Shengchao Wang, Yao Ye, Wenjie Sun, Haohua Lu, Zhi Ruan, Jinming Xu, Tianru Wang, Guang Lu, Liming Wang, Yu Shi, Honghe Zhang, Han Wu, Weiguo Lu, Han-Ming Shen, Dajing Xia, Yihua Wu

**Affiliations:** 1 Department of Toxicology of School of Public Health, and Department of Gynecologic Oncology of Women's Hospital, Zhejiang University School of Medicine, Hangzhou 310058, China; 2 Department of Gynecologic Oncology of Women's Hospital, Zhejiang University School of Medicine, Hangzhou 310058, China; 3 Department of Oncology, Second Affiliated Hospital, Zhejiang University School of Medicine, Hangzhou 310058, China; 4 Department of Pathology, Zhejiang University School of Medicine, Hangzhou 310058, China; 5 Sir Run Run Shaw Hospital, School of Medicine, Zhejiang University, Hangzhou 310016, China; 6 Department of Thoracic Surgery, The First Affiliated Hospital, Zhejiang University School of Medicine, Hangzhou 310003, China; 7 Epidemiology Stream, Dalla Lana School of Public Health, University of Toronto, M5T 3M7 ON, Canada; 8 Department of Physiology, Yong Loo Lin School of Medicine, National University of Singapore, Singapore 117597, Singapore; 9 State Key Laboratory for Diagnosis and Treatment of Infectious Diseases, Collaborative Innovation Center for Diagnosis and Treatment of Infectious Disease, The First Affiliated Hospital, Zhejiang University School of Medicine, Hangzhou 310003, China; 10 Second Affiliated Hospital, Zhejiang University School of Medicine, Hangzhou, 310009, China; 11 Faculty of Health Sciences, University of Macau, Macau, China

## Abstract

Autophagy is an essential cellular process that is closely implicated in diverse pathophysiological processes and a variety of human diseases, especially tumors. Autophagy is regarded as not only an anti-cancer process in tumorigenesis but also a pro-tumor process in progression and metastasis according to current research. It means the role of autophagy in tumor is considered to be complex, controversial and context dependent. Hence, a comprehensive database is of great significance to obtain an in-depth understanding of such complex correlations between autophagy and tumor. To achieve this objective, here we developed the Autophagy and Tumor Database (named as ATdb, http://www.bigzju.com/ATdb/#/) to compile the published information concerning autophagy and tumor research. ATdb connected 25 types of tumors with 137 genes required for autophagy-related pathways, containing 219 population filters, 2650 hazard ratio trend plots, 658 interacting microRNAs, 266 interacting long non-coding RNAs, 155 post-translational modifications, 298 DNA methylation records, 331 animal models and 70 clinical trials. ATdb could enable users to search, browse, download and carry out efficient online analysis. For instance, users can make prediction of autophagy gene regulators in a context-dependent manner and in a precise subpopulation and tumor subtypes. Also, it is feasible in ATdb to cluster tumors into distinguished groups based on the gene-related long non-coding RNAs to gain novel insights into their potential functional implications. Thus, ATdb offers a powerful online database for the autophagy community to explore the complex world of autophagy and tumor.

Database URL: http://www.bigzju.com/ATdb/#/

## Introduction

As a highly conserved cellular process, macroautophagy (hereafter referred to as autophagy) is essential for the degradation of cytoplasmic components to recycle intracellular substances so as to maintain the cellular homeostasis (1–5). Up to date, great advances have been made in deciphering the molecular mechanisms of autophagy, as well as in understanding the pathophysiological effects of abnormal autophagy in disease conditions such as tumor (2, 6, 7).

The autophagy process is controlled by the core autophagy machinery consisting of several key protein complexes encoded by key autophagy-related genes (‘ATGs’) and this machinery is regulated by several upstream signaling molecules and pathways such as mTORC1, AMPK, p53, TFEB, FoxO, etc. (1, 2). More importantly, both the core ATG proteins and their regulators are functionally modulated by various mechanisms, such as transcriptional regulation, post-translational modification (PTM), protein–protein interaction, non-coding RNA and chemical/pharmacological compounds (8–11). Another aspect of challenge comes from its complex effects on disease especially on tumor. Clinical trials using autophagy inhibitors, such as hydroxychloroquine (HCQ), have also reported diverse outcomes (12–15). Therefore, capture, compiling, annotation and analysis of such information are of great significance.

At present, several databases have been published to annotate autophagy-related literature. Autophagy Database and HADb (Human Autophagy Database) (16, 17) published in 2011 provided the glossary of ATGs and proteins. To elucidate the modulators and regulators of autophagy, ARN (Autophagy Regulatory Network), ncRDeathDB, THANATOS, HAMdb (Human Autophagy Modulator Database) and ACDB (Autophagic Compound Database) were also established (8–11, 18). Moreover, ATD (Autophagy To Disease) annotated the genes and chemicals, which are implicated in autophagy-associated human diseases (19).

Although the above-mentioned databases offer efficient tools to autophagy researchers, some important information is still absent. In particular, due to the complex role of autophagy in tumor (2, 20, 21), it is rather challenging to elucidate the detailed effects of autophagy using a ‘one size fits all’ strategy (22). More and more evidences were compiling to indicate multiple effects of autophagy. On the one hand, autophagy was reported to suppress tumor, for example, by suppressing SQSTM1/p62 gathering, inappropriate activation of NRF2, subsequent reactive oxygen species (ROS) activation and continuous inflammation (23, 24). On the other hand, autophagy facilitates tumor promotion and therapy resistance, for example, via resisting starvation, maintaining mitochondrial function assisting DNA repair and so on (23, 24). When it comes to cancer treatment by autophagy manipulation, final outcomes might be influenced by gene expression, non-coding RNA signature, clinical features, etc. Challenges and opportunities remain to identify the patients that are mostly likely to benefit from this autophagy modulation. Thus, a tool that explores the potential effects of autophagy in different situations is in great demand.

At present, there is no comprehensive database covering autophagy and tumor. Thus, we developed Autophagy and Tumor Database (ATdb, http://www.bigzju.com/ATdb/#/), a comprehensive database covering the literature of autophagy and tumor research. ATdb presents information about 137 genes required for autophagy-related pathways in 25 types of tumors. Users can get access to the informative data via the user-friendly interfaces.

## Materials and Methods

### Data source and collection

The Cancer Genome Atlas (TCGA) data from Xena GDC TCGA (https://xenabrowser.net/datapages/) were downloaded. The downloaded data included gene expression RNAseq, phenotype, survival data, miRNA expression quantification and transcript expression RNAseq.

In Genes Section, general information of a certain gene was collected from Human Protein Atlas, HGNC, UniProt and ENSEMBL. Official symbol, synonyms, Entrez ID, Ensembl ID and summary were collected from https://www.ncbi.nlm.nih.gov/gene/. External links for UniProt and HGNC were collected manually. Expression profiles and intracellular localization images were downloaded from Human Protein Atlas (25). Pearson correlation coefficient and *P*-values were calculated to assess the correlation between genes and other types of molecules. Literature searching and manual curation-assisted text mining were carried out to screen the experimentally identified PTMs, non-coding RNA regulations, DNA methylation and animal models about genes. Potential regulators, methylation records and animal models identified by experiment were searched in PubMed. Articles that reported any experimentally verified result of the above topics would be included. Two reviewers independently collected data using standardized forms and discrepancies were resolved by a third investigator. Search terms were listed in the Supplementary Table 1. RNA sequencing data from TCGA were adopted to predict possible interactions among genes and transcription factors, miRNAs and long non-coding RNAs (lncRNAs). Alternative splicing expression distribution was derived from TCGA data set. To identify genes required for autophagy-related pathways, PubMed was searched with ‘autophagy[ti] AND (mechanism[ti] OR gene[ti] OR genes[ti] OR molecule[ti] OR molecular[ti])’. Based on the consensus of all authors, four representative reviews on autophagy were selected to extract a list of genes. (1–3, 26) Finally, 137 genes were included. Then, experts in the field of autophagy research were consulted to review the list of the included genes.

**Table 1:** Basic statistics of the database

	Items	Number
ATG gene		
General information	Genes	137
	Cell lines	64
	Tissues	46
	Intracellular location images	1485
Regulation	miRNA	658
	lncRNA	266
	PTM	155
	Methylation	298
Animal models		331
Autophagy in Tumors		
Types of tumors		25
Significant ATG regulators	Transcription factor	11 288
	miRNA	356
	lncRNA	4149
Survival analysis	GEO data sets	93
	TCGA data sets	25
	Population filters	219
	HR trend images	2650
Clinical trials		70

For pages of Autophagy in Tumors, MeSH terms were extracted from https://www.ncbi.nlm.nih.gov/mesh/. Summaries were extracted from NCCN guidelines. Gene expression was calculated from TCGA gene expression RNAseq in R software with Linnorm package. Pearson correlation coefficient and *P*-values were calculated. The colors in heat plots gradually changed from red to blue, indicating the value of correlation coefficient ranging from −1 to +1. Expression data and survival data of 25 types of tumors from both TCGA and Gene Expression Omnibus (GEO) were integrated to predict tumor-specific genes regulation, to customize survival analysis and to calculate the differential expression genes. In customized survival analysis, population filters were applied to enable the selection of a specific population. Apart from TCGA data sets, 93 serials from GEO provide a broader view of survival analysis. Filters for population were extracted from TCGA phenotype data based on the knowledge of the tumors. The customized Kaplan–Meier plots were drawn from the TCGA survival data and GEO data sets. The hazard ratio (HR) trend plots were drawn in the R software with the code adopted from a published article (27). Clinical trials were retrieved from ClinicalTrials.gov (https://www.clinicaltrials.gov/).

Cluster analysis and Kaplan Meier plot in the results were generated with the R package factoextra, cluster, survival and survminer. The k-means clustering was employed.

### Database and website implementation

To store and maintain data, MySQL database was employed. The data were loaded into the MySQL server with mysqlimport client. In the MySQL database, 50 tables were created to store different information. The tables and correlation between tables were designed for convenient future update. The database will be continuously maintained and updated according to the same protocol that established in this study. All the code used in this study will be preserved for future update to minimize the heterogeneity.

Tomcat was the web server. On client request, JAVA was used to extract requested data from MySQL. JSON was the data transmission format between web server and client. JavaScript and jQuery on the client side were used to create interactive user interface. Google Chrome, Firefox and Safari were the recommended browsers.

**Figure 1:**
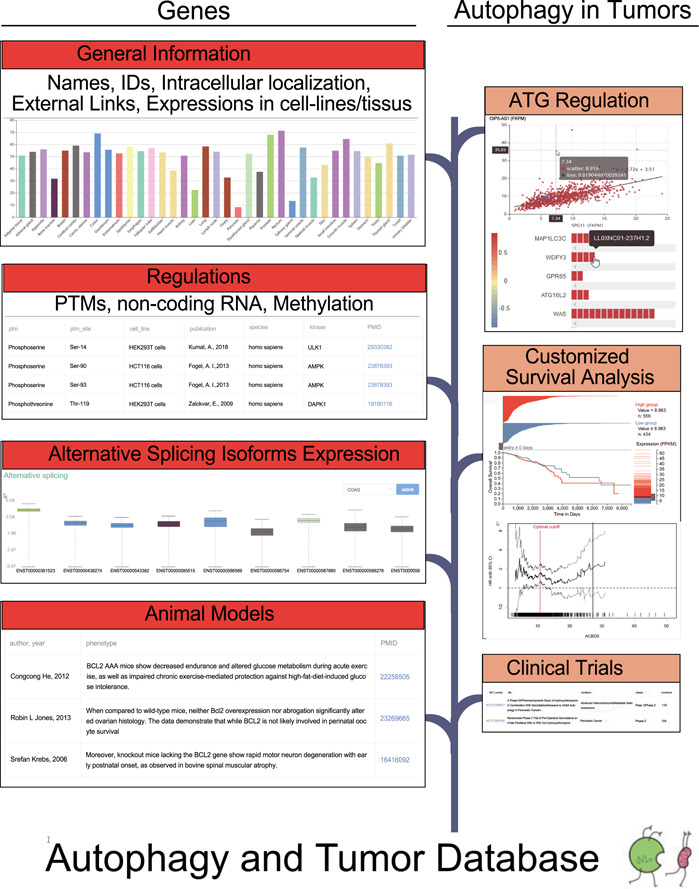
The flow diagram of the database construction. Gene-specific information was collected from Human Protein Atlas, HGNC, UniProt and Ensembl. Experiments identified regulators was searched in the PubMed. RNA sequencing data from TCGA were used to carry out correlation analysis and differential expression analysis. TCGA survival data and phenotype data were used to customize survival analysis.

## Results

### Contents of the ATdb

As a database dedicated to autophagy research, ATdb aims to extensively collect and visualize the known information of included genes and bridge the gap between autophagy and tumor research. From literature reviewing, we extracted a list of 137 genes (see Supplementary Table 2) required for autophagy-related pathways as cornerstone of ATdb (1–3, 26).

The basic statistics of the ATdb are listed in the Table 1. In the database, it currently includes 137 genes required for autophagy pathways, with expression profiles in 64 cell lines and 46 tissue types. To find experimentally identified results, manual curation was used to obtain 658 interacting miRNAs, 266 interacting lncRNAs, 155 PTMs, 298 DNA methylation records, 331 animal models and 70 clinical trials. The clinical effects of autophagy were analyzed in 25 types of tumors with data from TCGA and 93 Gene Expression Omnibus data sets. Among them, 219 population filters and 2650 HR trend plots were constructed to enable the customization of survival analysis. Based on these 137 genes, two parts, i.e. Genes Section and Autophagy in Tumors Section, were constructed as shown in the Figure 1. The flow diagram in Figure 1 illustrates the data collection procedure and the contents included.

**Figure 2:**
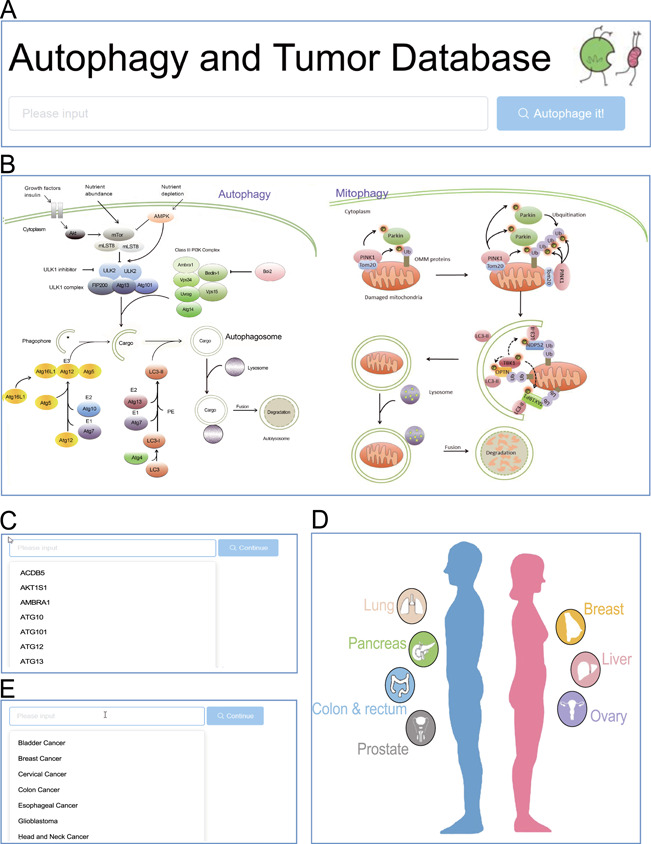
Browse entrance. (**A**) Search in the homepage; (**B**) click on the autophagy pathway illustration; (**C**) select from the dropdown list of genes; **(D)** click on the anatomy illustration; (**E**) select from the dropdown list of tumors.

Genes Section provided general information, regulatory factors, isoform expression level in different tumors and animal models. General information contained names, IDs, expression in cell lines/tissues and intracellular location. Previously published results of PTMs, nc-RNA regulations and gene methylation were extracted from literature. Correlation coefficients between ATGs and microRNAs/lncRNAs/transcription factors were calculated according to the expression levels and the results were visualized on the website. To facilitate autophagy research in tumor, Autophagy in Tumors Section was constructed. This section consists of the following subsections: a brief introduction of the selected tumor, gene expression differences among different situations, correlation analysis, customized survival analysis and relevant clinical trials.

**Figure 3:**
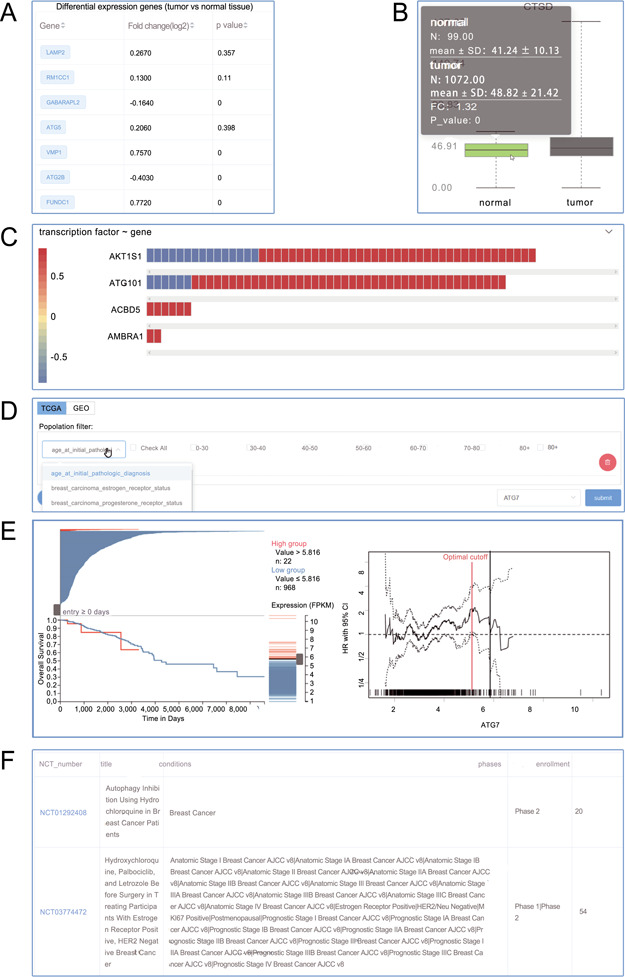
Screenshots from the Autophagy in Tumors. (**A**) Differential expression analysis; (**B**) box plot; (**C**) heat plot; (**D**) population filter for customized survival analysis; (**E**) interactive Kaplan–Meier plotter interface; (**F**) clinical trials.

### The ATdb website

ATdb is available at http://www.bigzju.com/ATdb/#/. The online website was developed to visualize the above-mentioned information with user-friendly interface. Data query, browsing, visualization and downloading were all fulfilled. ATdb mainly consists of four sections: search, genes, tumors and download.

The ATdb website provided multiple access to either a gene-specific page or tumor-specific page (Figure 1). The search box would attempt to auto-complete the search terms. Once the search term successfully matched with a preset entity, the website would navigate to the exact page that focuses on a certain gene or tumor (Figure 2A). Pathway- or anatomic-illustration and dropdown list would provide a better user experience (Figure 2B–E).

In the page dedicated for a particular tumor, introduction was followed by differentially differential expression (Figure 3A and B) and correlation analysis (3C). Notably, the ATdb provided easy-to-use customized survival analysis for each type of tumor. Based on medical knowledge, pre-built tumor-specific filters (Figure 3D) made it possible to accomplish the precise selection of patient population. The filter allowed users to define a combination of a subpopulation with a certain gene. This online analysis tool enabled dynamic Kaplan–Meier plotter with a dynamic cutoff point adjusted by a slider from TCGA and GEO data set (Figure 3E). Relevant clinical trials were displayed at the end (Figure 3F).

**Figure 4:**
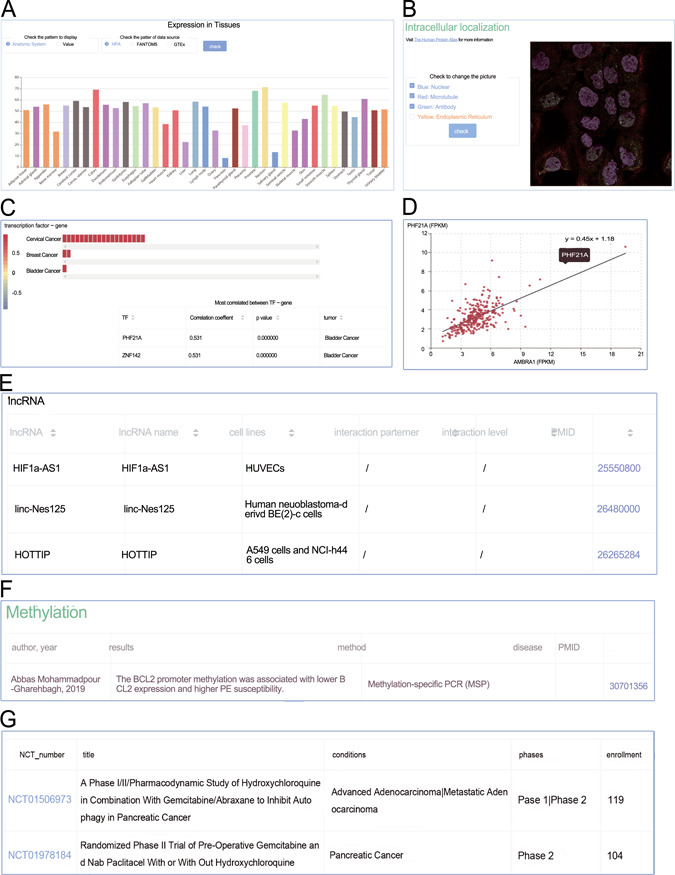
Screenshots from the genes section. (**A**) Expression profiles; (**B**) intracellular location; (**C**) heat plot; (**D**) scatter plot; (**E**) manual curated regulators; (**F**) DNA methylation; (**G**) animal models.

Similarly, the page of a certain gene and the general information with external links were placed at the top. Expression profiles (Figure 4A), intracellular localization (Figure 4B), correlation (Figure 4C) and scatter plot (Figure 4D) were clearly displayed sequentially. In the subsection Correlation, the expression correlations between the target gene and three kinds of categories (microRNA, lncRNA and transcription factor) were explored. Filters on tumor, absolute value of correlation coefficient and *P*-value were available. The colors of heat plots gradually changed from red to blue, indicating the value of correlation coefficient ranging from −1 to +1. Clicking on the colored cells in the horizontal bars would activate the scatter plot (Figure 4D). The following tables focused on the manually curated PTMs, microRNA regulations, lncRNA regulations (Figure 4E), experimentally verified DNA methylation (Figure 4F) and animal models (Figure 4G). Alternative splicing expression distribution in different tumors was also available.

The website allows customized data download via API. Detailed documents were in the Downloads.

### Comparison with other autophagy databases

In the past years, several autophagy-related online databases had been launched. Comparison between ATdb and pre-exist resources was carried out as shown in the Table 2. Autophagy Database provided a list of autophagy-related proteins and homologs in 41 eukaryotes (17). HADb integrated genomic data and sequence analysis features and displayed the information and sequences (16). ARN comprehensively collected predicted regulators from multiple sources and manually curated data to analyze the regulatory mechanisms of autophagy (11). The ncRDeathDB focused on ncRNA-associated cell death interactions in 12 species (10). THANATOS is a comprehensive database of PTMs associated with the regulation of autophagy (9). HAMdb archived the specific effects of proteins, chemicals and microRNA on autophagy and disease information (18). ATD annotated the genes and chemicals influencing autophagy and human diseases (19). ACDB compiled compounds that regulat autophagy (8).

**Table 2:** Comparison of databases

	Topic	Clinical trials	Survival analysis	Alternative splicing	Methylation	Animal model OR disease	General information	PTM	Homologs	Chemicals	Literature manual curation
Autophagy Database	Autophagy genes and proteins	−	−	−	−	−	+	−	+	−	−
HADb	Autophagy genes and proteins	−	−	−	−	−	+	−	+	−	−
ARN	Regulation	−	−	−	−	−	+	+	−	−	+
ncRDeathDB	Regulation	−	−	−	−	−	−	+	+	−	+
THANATOS	Regulation	−	−	−	−	−	+	+	+	−	+
HAMdb	Regulation	−	−	−	−	+	+	+	−	+	+
ATD	Diseases	−	−	−	−	+	−	+	−	+	+
ACDB	Compounds	−	−	−	−	+	−	−	−	+	+
ATdb	Tumors	+	+	+	+	+	+	+	−	−	+

Compared with the above resources, ATdb is unique in bridging tumor-autophagy research, customizing gene-related survival analysis in tumor, manually curating experimental results of DNA methylation and visualizing alternative splicing expression distribution in different tumors. Of note, ATdb features the compiling of *in vivo* results and population-based results such as animal models and clinical trials, which provides higher level of evidence for the designing of population-based studies. ATdb covers a broader range of fields in autophagy research and closely connects the autophagy research to its clinical significance in cancer.

### Applications

ATdb does not only provide an information stream but also clues future research. One feature of ATdb is its tight and efficient connection between molecules and tumors. Here, we demonstrated one of the possible applications of ATdb in clinical cancer research. Through the methods of correlation analysis and clustering analysis, patients were clustered into different groups and mostly linked molecules were detected.

**Figure 5:**
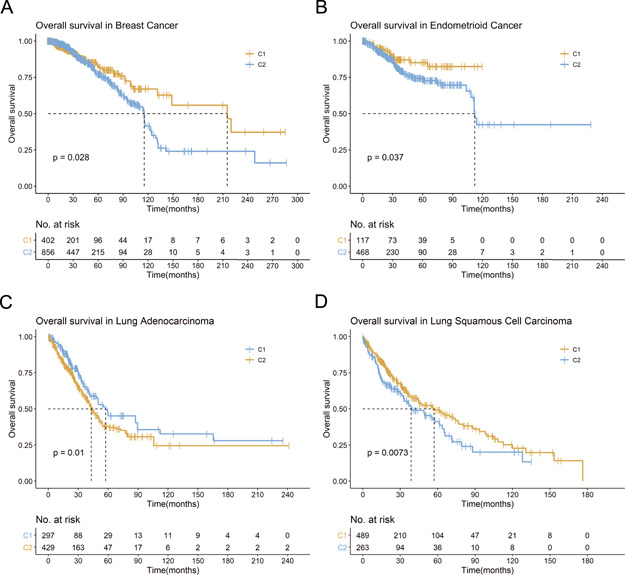
Example for ATdb application. Kaplan–Meier plot for (**A**) breast cancer (TCGA-BRCA); (**B**) endometrioid cancer (TCGA-UCEC); (**C**) lung adenocarcinoma (TCGA-LUAD); (**D**) lung squamous cell carcinoma (TCGA-LUSC).

Based on the included genes, TCGA breast cancer patients were clustered into two groups (Supplementary Figure 1A). Kaplan–Meier plot and log-rank test indicated statistically significant survival difference (Figure 5A). Similar results were observed in TCGA endometrioid cancer (Supplementary Figure 1B; Figure 5B). lncRNA has been shown to play a significant modulating role in the multistep biological processes of tumorigenesis. By employing the ATdb, we extracted significant correlated ($\Big|r\Big|$ > 0.7, *P* < 0.05) lncRNAs (Supplementary Table 3). Based on these lncRNAs, patients from TCGA-LUAD (TCGA Lung Adenocarcinoma) were clustered into two groups (Supplementary Figure 1C). Kaplan–Meier plot and log-rank test indicated statistically significant survival difference (Figure 5C). As shown in Supplementary Figure 1D and Figure 5D similar results were observed in TCGA-LUSC (TCGA Lung Squamous Cell Carcinoma). These results indicated that ATdb would facilitate tumor subtyping based on either genes or predicted lncRNAs.

**Figure 6:**
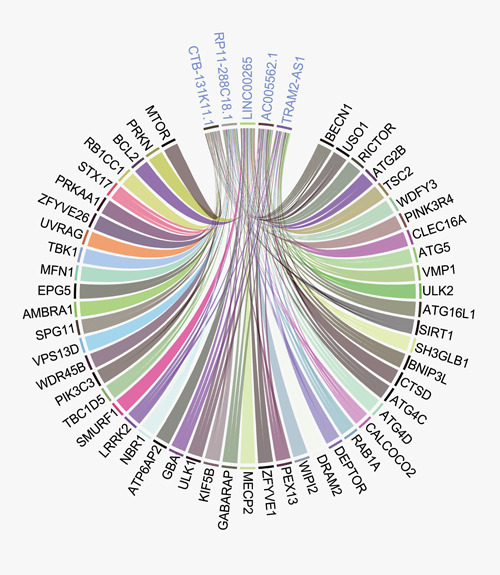
Circus plot between the most connected lncRNAs and genes. The top five most connected lncRNAs are connected with a wide range of genes.

To detect the most correlated lncRNAs and genes required for autophagy-related pathways, we counted all the significant links between them ($\Big|r\Big|$ > 0.7, *P* < 0.05). In total, 4149 links were detected in 25 types of tumors with 114 genes and 1097 lncRNAs. The number of links of each gene and lncRNA was counted. The number of related genes for 1097 lncRNAs was shown in the Supplementary Figure 2A. The Q95-Q100 lncRNAs were displayed in the Supplementary Table 4. The five most connected lncRNAs were found to be associated with a wide range of genes (Figure 6). A similar pattern was observed in the distribution of the number of related lncRNA for the included genes (Supplementary Figure 2B; Supplementary Table 5).

## Discussion

Autophagy is an essential event during tumorigenesis and tumor progression. (2, 22, 28–31) The controversial roles of autophagy triggered vast discussion. For example, to elucidate autophagy-mediated metastasis in a context-dependent nature, mechanisms and context underlying the role of autophagy in metastasis were reviewed by Dower *et al.* (20). Focal adhesion dynamics, integrin signaling and trafficking, Rho GTPase-mediated cytoskeleton remodeling, anoikis resistance, composition of the ECM, epithelial–mesenchymal transition signaling and tumor stromal cell interactions were thought to dictate the role of autophagy in metastasis (20). Other than macroautophagy, mitophagy, which is a specialized autophagy pathway, is emerging as a crucial player in modulating the response to therapies (29). Higher mitophagy levels protect the cancer cell from the damage caused by accumulating ROS and therefore contribute to therapy resistance. (29) Low mitophagy levels permit the apoptosis of cancer cell caused by lethal levels of ROS, thereby suppress tumor growth (29). To translate the known results into clinical practice, great progresses have been made in both clinical research and molecular epidemiology research. To test the effect of autophagy modulation in cancer treatment, a large number of clinical trials are on the way. HCQ, the widely autophagy inhibitor, and its use in cancer clinical trials were discussed in a review by Chude and Amaravadi (28). Other autophagy inhibitors, such as SBI-0206965, Spautin-1 and SAR405, are being developed (28). Moreover, several molecular epidemiology researches aim to clarify the relationship between autophagy and cancer prognosis. Niu *et al.* (32) established a scoring system based on pivotal ATG genes, which accurately predicts the prognosis advanced ovarian cancer patients. The ATG score may provide a novel perspective in applying autophagy into personalized therapy in the future.

However, precise and controllable autophagy manipulation is still waiting to be realized. Retrieval and integration of the published results are arduous. The online database closely connecting autophagy with cancer was missing. Hence, we presented the ATdb (http://www.bigzju.com/ATdb/#/), a practical tool with the comprehensive annotation and analysis on autophagy and tumor.

In the present study, ATdb connected 25 tumors with 137 genes required for autophagy-related pathways (Figure 1). To bridge the research between autophagy and tumor, 25 data sets from TCGA and 93 serials from GEO were incorporated. A total of 219 filters enabled users to assess the effects of autophagy in a certain population. Combined with gene expression, clinical stage, gender and other clinical phenotypes, a comprehensive and precise analysis could be carried out. ATdb could also facilitate tumor subtyping via new biomarkers. In this study, based on gene-correlated lncRNAs, lung adenocarcinoma and lung squamous cell carcinoma were both clustered into two groups with different survival outcomes. In addition, to interpret the biological functions of these 137 genes, we performed literature searching and manual curation-assisted text mining, which identified 658 interacting miRNAs, 266 interacting lncRNAs, 155 PTMs, 298 DNA methylation records, 331 animal models and 70 clinical trials. Alternative splicing isoform expression data across different tumors were visualized.

*In vivo* evidence and population-based evidence possess higher level of credibility because they are closer to clinical situation. Despite the confounding role of autophagy in disease treatment, autophagy manipulations in animals and populations, either by gene editing or medication, are already on the way. The key to success is to understand the effect of autophagy in tumorigenesis and tumor progression (22). This means further clinical trials should be built at least upon the existing evidence. ATdb integrated existing evidence of different autophagy-modulating drugs and tumors of different types, which could provide easier access to relevant information and facilitate further understanding of autophagy.

Although ATdb had already archived abundant information, ATdb has yet to be improved in several aspects. First, spectrum of included tumors should be extended. Second, information of some clinical trials is either incomplete or ongoing. Updating the information of these trials is necessary once they are completed. Third, mutations of autophagy genes might affect the tumorigenesis and such information is expected to be included once available. Finally, a survival analysis beyond gene expression (such as non-coding RNAs or methylation status) would provide additional crucial information and be beneficial to the improvement of our database.

In summary, we successfully established a comprehensive database ATdb on autophagy and tumor. ATdb captures the connections between genes required for autophagy-related pathways and tumor. Thus, it can serve as a useful tool toinspire and guide further studies on autophagy and tumor.

## Supplementary data

Supplementary data are available at *Database* online.

## *Conflict of interest*.

The authors declare no competing financial interests

## Supplementary Material

Supplementary_file_baaa052
