# Supplementary material for: Autophagy and Tumor Database: ATdb, a novel database connecting autophagy and tumor
Source: Database (Oxford). 2020 Jul 7;2020:baaa052. doi: 10.1093/database/baaa052 (PMC7340339; doi:10.1093/database/baaa052)
Supplement: Supplementary_file_baaa052 [file supplementary_file_baaa052.docx]

**Supplementary files**

**Autophagy and Tumor database: ATdb, a novel database connecting autophagy and tumor**

Kelie Chen^1#^, Dexin Yang^1#^, Fan Zhao^1#^, Shengchao Wang^2^, Yao Ye^3^, Wenjie Sun^4^, Haohua Lu^1^, Zhi Ruan^5^, Jinming Xu^6^, Tianru Wang^7^, Guang Lu^8^, Liming Wang^8^, Yu Shi^9^, Honghe Zhang^4^, Han Wu^10^, Weiguo Lu^2^, Han-Ming Shen^8*^, Dajing Xia^1*^, Yihua Wu^1*^

**Supplementary Figures**

**Supplementary Figure 1. Cluster Dendrograms.** Cluster Dendrogram for (**A**) breast cancer (TCGA-BRCA); (**B**) endometrioid cancer (TCGA-UCEC); (**C**) lung adenocarcinoma (TCGA-LUAD); (D) lung squamous cell carcinoma (TCGA-LUSC).

**Supplementary Figure 2. Distribution of the number of related genes for lncRNAs.** Barplot and density curve were drawn to display the distribution of the number of (**A**)related genes; (**B**) related lncRNAs.

**Supplementary Tables**

**Supplementary Table 1** Searching terms.

| topic | Search term |
| --- | --- |
| Animal model | (`gene_name`[tw]) AND (knockout[tw] OR knock?out[tw] OR mutant[tw] OR mutation*[tw] OR deficien*[tw]) AND (mouse[tw] OR mice[tw] OR murine[tw] OR fly[tw] OR flies[tw] OR zebrafish*[tw] OR worm*[tw]) |
| Methylation | (`gene_name`[tw]) AND (methylation[tw] OR methylated[tw] OR hypermethylation[tw]) |
| lncRNA | (`gene_name`) AND ((Noncoding RNA, Long) OR lncRNA OR (Long ncRNA) OR (ncRNA, Long) OR (RNA, Long Non-Translated) OR (Long Non-Translated RNA) OR (Non-Translated RNA, Long) OR (RNA, Long Non Translated) OR (Long Non-Coding RNA) OR (Long Non Coding RNA) OR (Non-Coding RNA, Long) OR (RNA, Long Non-Coding) OR (Long Non-Protein-Coding RNA) OR (Long Non Protein Coding RNA) OR (Non-Protein-Coding RNA, Long) OR (RNA, Long Non-Protein-Coding) OR (Long Noncoding RNA) OR (RNA, Long Untranslated) OR (Long Untranslated RNA) OR (Untranslated RNA, Long) OR (Long ncRNAs) OR (ncRNAs, Long) OR (Long Intergenic Non-Protein Coding RNA) OR (Long Intergenic Non Protein Coding RNA) OR (LincRNAs) OR (LINC RNA)) |
| microRNA | (`gene_name`) AND (MicroRNA OR miRNAs OR (Micro RNA) OR (RNA, Micro) OR miRNA OR (Primary MicroRNA) OR (MicroRNA, Primary) OR (Primary miRNA) OR (miRNA, Primary) OR (pri-miRNA) OR (pri miRNA) OR (RNA, Small Temporal) OR (Temporal RNA, Small) OR stRNA OR (Small Temporal RNA) OR pre-miRNA OR (pre miRNA)) |
| PTM | (`gene_name`)AND Phosphory* |

**Supplementary Table 2** List of 137 included genes.

| ACBD5 | GABARAPL2 | TBC1D15 | MEFV |
| --- | --- | --- | --- |
| AKT1S1 | HIF1A | TBC1D17 | MTMR3 |
| AMBRA1 | HSPD1 | TBC1D5 | PEX13 |
| ATG10 | KIF5B | TBK1 | PSEN1 |
| ATG101 | LAMP1 | TECPR1 | PTPN2 |
| ATG12 | LAMP2 | TFEB | RETREG1 |
| ATG13 | MAP1LC3A | TIMM23 | SLC33A1 |
| ATG14 | MAP1LC3B | TOMM20 | SMS |
| ATG16L1 | MAP1LC3B2 | TP53INP2 | SMURF1 |
| ATG16L2 | MAP1LC3C | TSC1 | SNX14 |
| ATG2A | MFN1 | TSC2 | SPG11 |
| ATG2B | MFN2 | ULK1 | TCIRG1 |
| ATG3 | MLST8 | ULK2 | TECPR2 |
| ATG32 | MTOR | ULK3 | TMEM230 |
| ATG4A | NBR1 | USO1 | VPS13D |
| ATG4B | NRBF2 | USP30 | WAS |
| ATG4C | OPTN | UVRAG | ZFYVE26 |
| ATG4D | PHB2 | VAMP8 |  |
| ATG5 | PIK3C3 | VDAC1 |  |
| ATG7 | PIK3R4 | VMP1 |  |
| ATG9A | PINK1 | WDFY3 |  |
| ATG9B | PLEKHM1 | WDR45 |  |
| BCL2 | PRKAA1 | WDR45B |  |
| BECN1 | PRKN | WIPI1 |  |
| BNIP3 | RAB11A | WIPI2 |  |
| BNIP3L | RAB1A | ZFYVE1 |  |
| CALCOCO2 | RAB24 | ZKSCAN3 |  |
| COX4I1 | RAB33B | APP |  |
| CTSB | RAB5A | ATP6AP2 |  |
| CTSD | RAB7A | C9orf72 |  |
| CTSL | RB1CC1 | CLEC16A |  |
| DEPTOR | RICTOR | CTNS |  |
| DNM1L | RPTOR | ERBB2 |  |
| DRAM1 | RUBCN | GBA |  |
| DRAM2 | SH3GLB1 | GPR65 |  |
| EPG5 | SIRT1 | GRN |  |
| FIS1 | SNAP29 | HTT |  |
| FUNDC1 | SQSTM1 | IRGM |  |
| GABARAP | STX17 | LRRK2 |  |
| GABARAPL1 | TAX1BP1 | MECP2 |  |

**Supplementary Table 3** Significantly correlated lncRNAs in LUAD and LUSC.

|  | lncRNA |
| --- | --- |
| LUAD | PCED1B-AS1, RP11-1334A24.6, RP11-750H9.5, USP30-AS1, AD000864.6, RP11-631N16.4, CTD-2002H8.2, RP5-1091N2.9, LINC01094, CARD8-AS1, RP11-121A8.1, TRG-AS1, RP11-760H22.2, FTX, LL0XNC01-237H1.2, CTD-2647L4.4, RP4-545K15.5, RP11-73M18.6, AC000123.2, NPTN-IT1, RP11-575L7.8, MIR600HG, RP11-295P9.3, LINC00174, SNHG12, LINC01089, SH3BP5-AS1, RP11-228B15.4, SEMA3F-AS1, ASMTL-AS1, LINC00106, RP5-890O3.9, LINC00893, RAD51-AS1, RP11-261P9.4, RP11-755F10.3, RP11-496I9.1, AP006621.6, RP11-521B24.5, RP11-66N24.3, AC009120.6, AC027601.1, LA16c-390E6.4, RP5-1142A6.9, RP11-264B17.4, LA16c-316G12.2, RP11-196G11.2, LA16c-358B7.3, RP11-334C17.5, CTC-479C5.10, CTC-524C5.2, RP1-59D14.5, RP11-68I3.4, RP11-1376P16.2, CTC-510F12.2, CTB-31O20.4, RP11-552F3.10, CTB-39G8.3, PTOV1-AS2, RP11-390K5.6, RP4-635E18.8, RP3-508I15.21, STAG3L5P-PVRIG2P-PILRB, RP3-402G11.27, RP11-347I19.8, RP3-402G11.28, RP3-402G11.25, RP13-516M14.10, RP11-449J21.3, RP11-20I23.2, AC006128.2, RP11-43N16.4, CTB-50L17.8, CTD-2013N17.6, CTBP1-AS, TEN1-CDK3, AC079630.4 |
| LUSC | C5orf56, PSMB8-AS1, RP11-24F11.2, LINC01272, RP5-1091N2.9, RP4-728D4.2, PCED1B-AS1, RP11-1334A24.6, RP11-750H9.5, USP30-AS1, RP11-219E7.1, RP11-284N8.3, RP11-389C8.2, RP11-473M20.9, RP11-848P1.3, AC006129.2, LLNLR-470E3.1, RP11-93B14.10, RP11-93B14.9, AD000864.6, TRG-AS1, SFTA1P, TBX5-AS1, CTD-2562J17.6, LINC01094, CARD8-AS1, RP5-899E9.1, RP11-760H22.2, SEMA3F-AS1, AC093110.3, HHIP-AS1, RBPMS-AS1, RP11-672A2.4, RP11-541N10.3, PCAT19, RP3-523K23.2 |

**Supplementary Table 4** List of the most connected lncRNAs.

| lncRNA | number ofconnected genes |
| --- | --- |
| AC005562.1 | 28 |
| CTB-131K11.1 | 24 |
| LINC00265 | 21 |
| RP11-288C18.1 | 21 |
| TRAM2-AS1 | 20 |
| LINC01128 | 19 |
| RP11-164P12.4 | 19 |
| EBLN3 | 18 |
| DYNLL1-AS1 | 16 |
| RTCA-AS1 | 16 |
| AC000123.2 | 15 |
| AC007879.6 | 15 |
| AC016747.3 | 15 |
| CITF22-24E5.1 | 15 |
| RP11-156P1.3 | 15 |
| RP11-342K6.2 | 15 |
| RP11-447D11.3 | 15 |
| USP46-AS1 | 15 |
| CTD-2015H6.3 | 14 |
| FGD5-AS1 | 14 |
| HCG18 | 14 |
| PAXIP1-AS2 | 14 |
| RP11-2N1.2 | 14 |
| RP11-353K11.1 | 14 |
| RP5-994D16.9 | 14 |
| AC006547.13 | 13 |
| CTD-2647L4.4 | 13 |
| CTD-3157E16.1 | 13 |
| LINC00674 | 13 |
| LINC01011 | 13 |
| LL0XNC01-237H1.2 | 13 |
| RP11-644F5.11 | 13 |
| TRAF3IP2-AS1 | 13 |
| CTC-351M12.1 | 12 |
| CTD-2083E4.7 | 12 |
| PCBP1-AS1 | 12 |
| RAP2C-AS1 | 12 |
| RP1-60O19.1 | 12 |
| RP11-701H24.3 | 12 |
| CTC-429P9.5 | 11 |
| OLMALINC | 11 |
| RP11-333E1.1 | 11 |
| LINC01272 | 10 |
| MKLN1-AS | 10 |
| RP1-239B22.5 | 10 |
| RP11-667K14.3 | 10 |
| WAC-AS1 | 10 |
| AC093642.5 | 9 |
| AC099850.1 | 9 |
| AF127936.9 | 9 |
| C15orf59-AS1 | 9 |
| CTC-429P9.2 | 9 |
| LINC00630 | 9 |
| LINC00957 | 9 |
| LINC01503 | 9 |
| PCED1B-AS1 | 9 |
| RASSF8-AS1 | 9 |
| RP11-10N23.2 | 9 |
| RP11-400F19.6 | 9 |
| RP11-43F13.3 | 9 |
| RP11-458J1.1 | 9 |
| RP11-53O19.1 | 9 |
| RP11-627K11.6 | 9 |
| RP11-6N17.4 | 9 |
| RP5-1061H20.4 | 9 |

**Supplementary Table 5** List of the most connected genes.

| gene | the numer of related lncRNAs |
| --- | --- |
| ATG16L2 | 389 |
| VMP1 | 153 |
| LRRK2 | 149 |
| GABARAP | 129 |
| RICTOR | 90 |
